# Hair Washes

**Published:** 1859-12

**Authors:** 


### HAIR WASHES.

There are only two which are always safe, and always efficient, cold water and soap-suds; the cold water once a day, the soap-suds once a week.

About two years ago, it was established in a court of justice in New York City, that one of the best, as well as one of the most popular hair washes, was simply soap-suds colored and scented; any one ingredient could be left out, except the soap-suds.

The most universally applicable treatment of the hair of boys, girls, and men, is as follows:—

Make half a pint of soap-suds with pure white soap and warm water, on rising any morning; but before applying it, brush the whole scalp well while the hair is perfectly dry, with the very best Russia bristle brush, scrub back and forth with a will, let not any portion of the surface escape. When brush ing the top and front, lean forward, that the particles may fall. After this operation is finished, strike the ends of the bristles on the hearth, or on a board, next pass the coarse part of the comb through the bristles; next, brush or flap the hair back and forth with the hand, until no dust is seen to fall; then with the balls of the fingers dipped in the soap-suds, rub the fluid into the scalp and about the roots of the hair; do this patiently and thoroughly; finally, rinse with clear water, and absorb as much of the water from the hair as possible with a dry cloth, then (after allowing the hair to dry a little more by evaporation, but not to dry entirely,) dress it as usual, always, under all circumstances, passing the comb through the hair slowly and gently, so as not to break any one off, or tear out any one by the roots.

By this operation, the alkali of the soap unites with the natural oil of the hair, and leaves it perfectly clean and beautifully silken, and with cold water washings of the whole head, and neck, and ears every morning, it will soon be found that the hair will "dress" as handsomely as if "oiled to perfection;" with the great advantage of conscious cleanliness, giving too, the general appearance of a greater profusion of hair than when it is plastered flat on the scalp, with variously scented hog's fat, as is the common custom.

There is a general saying, that cold water "rots the hair." The statement is of itself absurd. The hair is rotted by the filth which is allowed to cake upon the scalp by virtue of the grease, natural and artificial, gathering dust of every description, and making a composition, the very thought of which is nauseating.

Every mother who would pride herself in having her daughter possess a beautiful head of hair, luxurious, long and silken at sweet nineteen, should forbid any application to the hair, except pure water as above, keeping it short, and allowing it to lie naturally on the forehead.

